# Epidermolysis bullosa acquisita as an adverse effect from rituximab therapy: A case report

**DOI:** 10.1097/MD.0000000000023496

**Published:** 2020-12-04

**Authors:** Xueqiong Wu, Zhenhui Lv, Wenjia Li, Zhaosheng Meng, Shaw P. Wan

**Affiliations:** a Zibo Central Hospital; b The People's Hospital of Huantai County, Zibo, Shandong, China.

**Keywords:** adverse effect, case report, EBA, rituximab

## Abstract

**Rationale:**

Rituximab is a monoclonal antibody directed against B cells and is a first-line agent for the treatment of B cell lymphoma and a second-line agent for the treatment of idiopathic thrombocytopenic purpura (ITP). It has also been used for the treatment of several other autoimmune diseases. Epidermolysis bullosa acquisita (EBA) has never been reported as an adverse effect resulted from rituximab therapy.

**Patient concerns:**

A 54-year-old female presented with relapse of the ITP for around eight months. She was treated with rituximab. Intramuscular chlorpheniramine and intravenous methylprednisolone and cimetidine were used as premedication before rituximab infusion. The infusion was initially started at 50 mg/h for 1 h followed by 100 mg/h till the end of infusion. The day after rituximab infusion, the patient noticed pruritic blisters on both arms and chest skin. The next day, the lesions increased in severity and extent.

**Diagnosis:**

The skin biopsy established the diagnosis of EBA. H&E staining revealed subepidermal blisters infiltrated by inflammatory cells, including eosinophils and lymphocytes. Direct immunofluorescence (DIF) showed linear deposition of IgG and C3 at the dermoepidermal junction. Indirect immunofluorescence with the patient's serum on salt-split skin revealed exclusive dermal binding of circulating IgG antibasement membrane antibodies at a titer of 1:160.

**Interventions:**

She was treated with intravenous methylprednisolone and was continued on oral prednisolone.

**Outcomes:**

The lesions regressed. Six weeks later, she had a recurrence of similar lesions but in milder form. This episode subsided in 4 to 5 days with topical steroid application.

**Lessons:**

Physicians should consider this diagnosis when a patient develops bullous skin eruptions while undergoing Rituximab therapy.

## 1 Introduction

Rituximab, a chimeric monoclonal antibody directed against the CD20 antigen, is commonly used for the treatment of B-cell lymphoma.^[1,2]^ It is a second line regimen for the treatment of idiopathic thrombocytopenic purpura (ITP).^[3]^ Infusion reactions such as flushing, itching, dyspnea, heartache, fever, and nausea are the most common adverse effects of rituximab.^[4,5]^ Rituximab is also associated with several dermatologic reactions.^[6]^ Furthermore, cytokine release during the intravenous infusion of rituximab can cause urticaria and angioedema.

Epidermolysis bullosa acquisita (EBA) has never been reported in patients undergoing rituximab therapy. We herein report a case of EBA in a patient with ITP. In the present case, the condition developed during the first cycle of rituximab therapy. The patient has given a written informed consent for reporting this case.

## 2 Case report

A 54-year-old female presented with relapse of the ITP for around 8 months. She was diagnosed to have ITP about 10 years earlier. She was treated with prednisolone and cyclosporine. Serum complements were normal, and anti-dsNA antibody was negative. However, despite adequate trough levels of cyclosporine (C0 level maintained >100 ng/mL), there was no response after 3 months of treatment, which manifested by persistent thrombocytopenia. In view of the failed therapy, further treatment options were discussed, and an informed decision was made to use rituximab. Patient was counselled regarding its efficacy and adverse effects. Rituximab monotherapy with 375 mg/m^2^ was planned. Intramuscular chlorpheniramine and intravenous methylprednisolone and cimetidine were used as premedication before rituximab infusion. The infusion was initially started at 50 mg/h for 1 h followed by 100 mg/h till the end of infusion.

**Figure 1:**
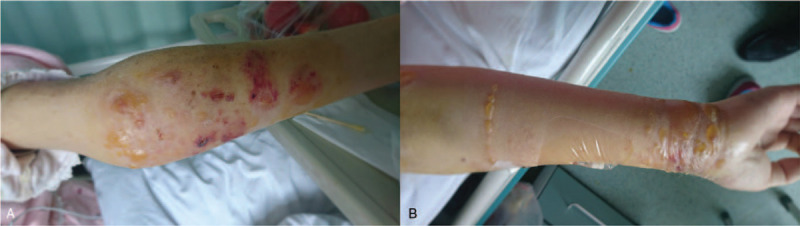
Lesions with clear blister fluid predominantly involving flexor and extensor aspects of bilateral upper limbs and chest skin.

The day after rituximab infusion, the patient noticed pruritic blisters on both arms and chest skin. On examination, these were lesions with clear fluid predominantly involving the skin of the flexor and extensor aspects of both upper limbs and chest (Fig. 1). The next day, the lesions increased in severity and extent.

**Figure 2:**
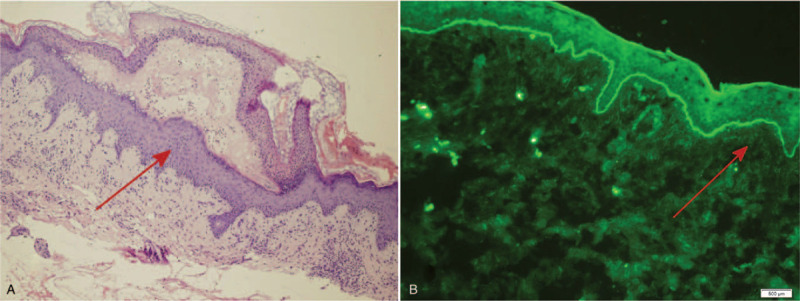
H&E staining and direct immunofluorescence of the skin biopsy.

After a skin biopsy, she was treated with intravenous methylprednisolone (according to prednisone 1 mg/kg·d) and the lesions regressed. The skin biopsy established the diagnosis of EBA (Fig. 2). H&E staining revealed subepidermal blisters infiltrated by inflammatory cells, including eosinophils and lymphocytes. Direct immunofluorescence (DIF) showed linear deposition of IgG and C3 at the dermoepidermal junction. Indirect immunofluorescence with the patient's serum on salt-split skin revealed exclusive dermal binding of circulating IgG antibasement membrane antibodies at a titer of 1:160.

She was continued on oral prednisolone (prednisone minus one tablet per week). Six weeks later, she had a recurrence of similar lesions but in milder form. This episode subsided in 4 to 5 days with topical steroid application (double the current dose of prednisone).

## 3 Patient perspective

This patient was promptly informed of this previously unknown adverse event. Treatment options were discussed. She was pleased that the skin lesions were an adverse effect from the medication rather than a new illness. She decided to follow our treatment recommendations and was very satisfied with the result.

## 4 Discussion

Rituximab is extensively used for the treatment of B cell lymphoma and as one of the leading second-line drugs in the treatment of ITP.^[3]^ It has also been used for the treatment of several other autoimmune diseases, including EBA.^[7]^ The side effects of this monoclonal antibody however remain a concern.

Walewski reported that 60% of the first, and 20% of subsequent rituximab infusions were associated with infusion-related reactions in patients with recurrent indolent lymphoma.^[4]^ These adverse effects included mild fever, chills, and occasional skin eruptions. Brown BA reported that infusion-associated events occurred in 25.7% of the multiple sclerosis patients. Reactions were mild-to-moderate; most occurred during the first infusion and most patients went on to receive subsequent doses without any further problems.^[8]^ Errante D reported urticarial reaction occurring 1 h after the start of retuximab infusion which spontaneously resolved after temporary stoppage of infusion. They hypothesized that the urticarial reaction in this particular patient with primary cutaneous B-cell lymphoma was an epiphenomenon of the killing of the B-cells bearing the CD20 antigen in the sites of the disease. This resulted in local cytokine release rather than the occurrence of a hypersensitivity reaction causing the urticarial reaction.^[9]^ In addition to infusion reactions, serum sickness is reported in 1% to 20% of patients, more commonly among those with autoimmune conditions.^[5]^ Diffuse, pruritic, maculopapular rash with generalized edema, associated with aphthous ulcers, diarrhea, arthralgias, and myalgia occurring 48 h after rituximab injection was described by Hellerstedt and Ahmed in a patient with systemic lupus erythematosus.^[10]^ Severe reactions including Stevens-Johnson syndrome and vasculitis have also been observed.^[11,12]^

EBA is a chronic autoimmune subepidermal blistering disease characterized by circulating and tissue-bound autoantibodies targeting type VII collagen, which is the major component of anchoring fibrils. EBA usually manifests as blisters and erosions on the skin and mucocutaneous areas. EBA is a rare disease. The overall prevalence has yet to be determined. EBA occurs in both genders, at all ages, and in all ethnic groups. Blisters appears to form either through a direct pathway, where the adhesive function of the basement membrane is impaired (mechanobullous type); or through an indirect pathway which involves the complement activation and neutrophil recruitment (inflammatory type).^[13]^

EBA has never been reported as a side effect with rituximab treatment. The mechanism for the occurrence remains unclear. Our patient developed the inflammatory type EBA. We posited that it might represent a delayed type of hypersensitivity reaction. The condition developed the day after rituximab infusion and she had not received any other new drugs. Hence, rituximab was thought to be the likely etiological agent rather than EBA being a coincidental occurrence. In addition, this patient has ITP, a known autoimmune disease, which might make her more susceptible to develop EBA. Fortunately, this patient responded well to steroid therapy.

Laboratory tests to diagnose EBA include H&E staining, immunofluorescence, immunoblotting, immunoelectron microscopy, and ELISA. Biopsy specimens of inflammatory EBA reveal subepidermal blisters infiltrated by various inflammatory cells, including neutrophils, eosinophils, and lymphocytes; whereas those from the mechanobullous EBA often show sparse infiltrate. Histopathological findings alone, cannot confirm a diagnosis of EBA because other subepidermal bullous diseases can have nearly identical appearance. Immunofluorescence studies are necessary to obtain a definitive diagnose. DIF shows linear deposition of IgG, C3, or both along the basement membrane zone. U-serrated immunodeposition pattern in DIF can help to differentiate EBA from other subepidermal bullous disease.^[13]^

Our patient was diagnosed to suffer EBA based on the histopathological findings and the immunofluorescence results.

## 5 Conclusion

EBA has not been previously reported as an adverse effect associated with rituximab therapy. With increased indications for the use of rituximab, it is imperative for clinicians to identify and manage the complications associated with this monoclonal antibody. This case highlights the importance of watching out for blister dermatitis as an early manifestation of rituximab toxicity. Patients should be educated to report any skin lesion that occurs during rituximab therapy; for it has the potential to evolve into more severe lesions if further doses are given.

## Acknowledgments

The authors [Xueqiong Wu, MD.; Zhenhui Lv, MS.; Wenjia Li, BS.; Zhaosheng Meng, BS.; Shaw P. Wan, MD.] organized the order of the visits of this patient and interpreted the results.

All authors approved the final manuscript as submitted and agree to be accountable for all aspects of the work. The authors received no financial compensation for this case report.

## Author contributions

**Formal analysis:** Wenjia Li.

**Resources:** Zhenhui Lv.

**Supervision:** Zhaosheng Meng.

**Writing – original draft:** Xueqiong Wu.

**Writing – review & editing:** Shaw P. Wan.
